# Life: Computational Genomics Applications in Life Sciences

**DOI:** 10.3390/life11111211

**Published:** 2021-11-09

**Authors:** Yuriy L. Orlov, Anastasia A. Anashkina

**Affiliations:** 1 The Digital Health Institute, I.M. Sechenov First Moscow State Medical University of the Ministry of Health of the Russian Federation (Sechenov University), 119991 Moscow, Russia; nastya@eimb.ru; 2 Life Sciences Department, Novosibirsk State University, 630090 Novosibirsk, Russia; 3 Agrarian and Technological Institute, Peoples’ Friendship University of Russia, 117198 Moscow, Russia; 4 Engelhardt Institute of Molecular Biology, Russian Academy of Sciences, 119991 Moscow, Russia

This Special Issue, “Life: Computational Genomics”, presents research articles on systems biology applications, computational genomics, and bioinformatics methods in life sciences. The molecular mechanisms of human disease progression, as well as gene expression in laboratory animal models are studied now based on sequencing technologies and genotyping tools. This Special Issue continues the collection of papers that were published in MDPI IJMS journal “Bioinformatics of Gene Regulations and Structure” (https://www.mdpi.com/journal/ijms/special_issues/Bioinformatics_Genomics, assessed on 8 November 2021) and “Medical Genetics, Genomics and Bioinformatics” (https://www.mdpi.com/journal/ijms/special_issues/Medical_Genetics_2021, accessed on 8 November 2021) [1,2], initiated after the series of bioinformatics conferences that were held in Russia–BGRS (https://bgrssb.icgbio.ru/2020/, assessed on 8 November 2021) and the Symposium on Bioinformatics and Computer-Aided Design of Drugs (http://way2drug.com/dr/symp_bcadd_2021_en.php, assessed on 8 November 2021). The presented works were reported in part at the BGRS (Bioinformatics of Genome Regulation and Structure/Systems Biology) traditional biannual computational biology meeting in Novosibirsk, highlighting recent advances in genomics, bioinformatics, systems biology, and biotechnology areas. This collection continues the studies in the field of bioinformatics that were presented initially in the *Frontiers in Genetics* journal [3], in the *PeerJ* journal (https://peerj.com/collections/72-bgrs-sb-2020, assessed on 8 November 2021) [4], and recently at MDPI *International Journal of Molecular Sciences* [2] (https://www.mdpi.com/journal/ijms/special_issues/Medical_Genetics_Bioinformatics, assessed on 8 November 2021). This Special Issue is related to the topics on computational genomics in model organisms for biotechnology, discussing genomics and bioinformatics approaches for life sciences. 

The series of post-conference Special Issues started with a coverage of Bioinformatics of Genome Regulation and Structure (BGRS) [5,6,7] conferences and related Schools on Systems Biology and Bioinformatics (SBB) held in Novosibirsk, Russia [8], later completed by other international conferences on genetics such as Belyaev Conference–2017 [9,10,11]. The papers in this issue present novel bioinformatics tools, animal genomics application, and the genetics models of human diseases. 

We open this collection of papers by Bo Song et al. [12] presenting a novel bioinformatics tool for ribosome profiling (Ribo-seq) analysis. Ribo-seq technology extended our knowledge about translation in wide range of organisms [13]. The RiboNT tool, a noise-tolerant open reading frames (ORF) predictor can utilize ribosome-protected mRNA fragments with poor periodicity.

The next group of papers presents applications in animal genomics. Alexander Igoshin et al. [14] considers problems of cold tolerance in Siberian cattle. The authors discuss the chromosomal regions and candidate SNPs (single nucleotide polymorphism) controlling the body temperature of Siberian cattle populations. Some genes that were found were previously known in relation to thermal adaptations in cattle and other species. Advances in genetic tools that have been applied to livestock breeding had prompted research into the adaptation of the breeds to climate and local environments [15]. Animal genes that are putatively related to environmental adaptations were studied in [16] and presented in previous special post-conference journal issues. The problems of genomics in pigs that are related to breeding are presented in [17,18] by the group of Prof. Lyubov Getmatseva. The authors had studied genomic regions and genes linked to the capped hock in pigs [17] using genotyping. Siroj Bakoev et al. [18] studied genome homozygous segments in purebred and crossbred pigs. An accumulation of homozygosity can lead to an increase in inbreeding and the accumulation of deleterious variants, presenting an economical problem in farming. Using sequencing technology this group recently studied mitochondrial DNA and genome diversity in Large White pigs in Russia [19,20].

Evgeniya Poltavskaya et al. [21] presented study of gene polymorphism in humans that is related to mental disorders and schizophrenia. The authors examined the effects of GRIN2A and GRIN2B (Glutamate Ionotropic Receptor NMDA Type Subunit 2A/2B) polymorphisms on the clinical features of schizophrenia using SNPs genotyping. Note that this approach has been used for gene polymorphism studies in association with alcohol use disorder [22] and complex disorders such as metabolic syndrome [23] that are related to *FTO* (fat mass and obesity-associated) gene. Associations of the *FTO* gene polymorphic variants were in shown [23]. Note that the genetic component in the *FTO* gene is related to high obesity risk for in human populations [24]. These medical genomics problems were discussed at BGRS conference and presented in special post-conference journal issues at *BioMed Central* [6,7]. 

The analysis of genetics backgrounds and pathways in complex diseases such as schizophrenia, metabolic syndrome, and autism spectrum disorders demands the development of genomics and system biology methods [25,26]. The analysis of gene network structure and the annotation of hub genes in disease networks may suggest new targets for therapy [27].

Priyanka Upadhyai et al. [28] discussed the actual problem of genetic background in the coronavirus disease severity in populations. The authors studied variances in the hosts genetic architecture that may underlie the inter-individual and population-scale differences in COVID-19 manifestation using genotyping data from the AncestryDNA database (https://www.ancestry.com/dna/, assessed on 8 November 2021). The distinct genetic variants were found to be associated with pathways that govern host immunity, such as interferon, interleukin, and cytokine signalling, as well as known COVID-19 comorbidities, such as obesity and cholesterol metabolism [29].

Overall, we are proud of the continuing Life Special Issue that we have collated together as well as *IJMS* special issue on bioinformatics of gene regulation [30]. We hope that you will find this collection of papers stimulating reading, consider coming to the next BGRS\SB conferences in Novosibirsk, Russia (https://bgrssb.icgbio.ru/2022/, assessed on 8 November 2021), and consider the next special journal issues on the genomics technologies applications at MDPI IJMS “Plant Biology and Biotechnology: Focus on Genomics and Bioinformatics” (https://www.mdpi.com/journal/ijms/special_issues/Plant_Biotechnology, assessed on 8 November 2021), as well as “Molecular Mechanisms of Transcriptional Regulation in Tumor Cell” at Genes journals (https://www.mdpi.com/journal/genes/special_issues/Transcriptional_Regulation_Tumor, assessed on 8 November 2021).
